# Beverage of Brazil Nut and Bocaiuva Almond Enriched with Minerals: Technological Quality and Nutritional Effect in Male Wistar Rats

**DOI:** 10.3390/foods13162533

**Published:** 2024-08-14

**Authors:** Bianca Ribeiro de Oliveira, Mariana Buranelo Egea, Suélem Aparecida de França Lemes, Thais Hernandes, Katiuchia Pereira Takeuchi

**Affiliations:** 1 Instituto Federal de Educação, Ciência e Tecnologia Goiano—IF Goiano, Campus Rio Verde, Rio Verde 75901-970, GO, Brazil; biancaribeiro.oliveira@gmail.com; 2 Departamento de Química, Universidade Federal de Mato Grosso—UFMT, Campus Cuiabá, Cuiabá 78060-900, MT, Brazil; suelem.nutri@gmail.com; 3 Departamento de Alimentos e Nutrição, Universidade Federal de Mato Grosso—UFMT, Campus Cuiabá, Cuiabá 78060-900, MT, Brazil; thais.hernandes@ufmt.br

**Keywords:** plant-based milk, supplementation, triglycerides, heat treatment

## Abstract

The objective of this work was to evaluate the properties of beverages formulated with Brazil nuts (*Bertholletia excelsa* Bonpl) and bocaiuva almonds (*Acrocomia aculeata* (Jacq.) Lodd. Ex Mart.). Five beverages were developed with Brazil nut, bocaiuva almond, and water (*m*/*m*/*v*), as follows: (i) NB, nut:water, 1:10; (ii) AB, almond:water, 1:10; (iii) NAB1, 1:0.1:10, nut:almond:water; (iv) NAB5, nut:almond:water, 1:0.5:10; and (v) NAB10, nut:almond:water, 1:1:10. The physicochemical, chemical, technological, and microbiological parameters were evaluated. After heat treatment (HT) and enrichment with minerals, the beverages that demonstrated stability in these characteristics were tested in a biological assay. The physical and biochemical parameters of male Wistar rats were evaluated after administering beverages for 28 days. HT decreased the total phenolic content and the antioxidant activity; however, it guaranteed microbiological safety. Mineral supplementation changed the colors and increased the pH values of the beverages. After the beverages were administered, the Wistar rats in the (i) NB group showed decreases in retroperitoneal adipose tissue, total cholesterol, and triglycerides; (ii) those in the AB group exhibited decreased triglycerides contents; and (iii) those in the NAB10-group presented decreased liver weights. The beverages evaluated in this study demonstrate a protective effect against risk factors such as fat accumulation in the liver, retroperitoneal adipose tissue, and hypercholesterolemia.

## 1. Introduction

Over 2 billion people worldwide do not have regular access to safe, nutritious, and sufficient food [1]. The World Health Organization (WHO) estimates that approximately half of the 1.62 billion cases of anemia worldwide are due to iron deficiency [2]. In 2011, 3.5 billion people were at risk of calcium (Ca) deficiency due to inadequate dietary supply [3]. Calcium is required for bone-forming, vascular contraction, vasodilation, muscle function, nerve transmission, intracellular signaling, and hormonal secretion. Less than 1% of the body’s total calcium is needed to support these critical metabolic functions, but their role is indispensable for maintaining health, especially bone formation [4]. In this way, the production of fortified/enriched foods has grown to meet people’s unique needs [5].

Producing beverages from water-soluble extracts of plant sources has been an adopted strategy due to their low cost and good nutritional quality [6]. In addition, consumers consider plant-based beverages as an alternative to cow’s milk (considered a source of calcium) for persons with lactose intolerance or milk allergy [7].

In addition to plant-based beverages having low calcium and/or iron, the presence of phytates in seeds and the application of heat treatment can also reduce the bioavailability of the micronutrients. Thus, plant-based beverage supplementation can be used as an alternative to improve the nutritional value [8]. However, mineral supplementation may affect the acidification or alkalization in matrix interactions, altering the stability of beverages, and pH changes dramatically modify food products’ sensory properties and microbiological safety [9].

Extraction technologies in beverage production may also improve sensory characteristics, with improvement in their potential market through adding additives, ingredients, or other raw materials, which result in different tastes and aromas [6]. Many preservation methods (thermal and nonthermal) are used to increase these beverages’ stability during their shelf life. However, the effects of the method on technological characteristics must be studied in each beverage [8]. In addition to mineral enrichment, extraction methods can modify the technological characteristics during the beverage’s shelf life [10].

*Bertholletia excelsa* Bonpl. is a species native to the Amazon region. Brazil nut, as it is commonly known, is a nutritious food with high energy and contains high protein, carbohydrate, polyunsaturated lipid, vitamin, and mineral contents, especially selenium, as well as phenolic and antioxidant compounds [11]. Brazil nuts are harvested in the country’s interior and marketed primarily in cooperatives and small industrial organizations, where thermal processing for beverage production is predominantly utilized [11,12]. Meanwhile, the *Acrocomia aculeata* (Jacq.) Lodd. Ex Mart. fruit—a Midwestern Brazilian plant known as bocaiuva—contains edible pulp, also used for juices and desserts [12,13], and almonds that represent 48% of the fruit’s total weight. Although this almond shows potential for use in several areas, such as culinary preparations, a supply of nutritional elements, cosmetics, and energy production [13,14], bocaiuva almond is still unknown by the population. 

Although some research has demonstrated a potential beneficial effect in vitro due to bioactive compounds in Brazil nut and bocaiuva almond, little is known about the actual beneficial effect in vivo, especially after heat treatment application to the beverage. Therefore, we hypothesized that beverages produced with Brazil nut and bocaiuva almond and supplemented with minerals (calcium and iron) could change the physical and biochemical characteristics of Wistar rats (28 days of administration). As we used new (i.e., noncommercial) beverages, we tested several conditions, such as heat treatment and different proportions of Brazil nut and bocaiuva almond. We also evaluated the physicochemical characteristics of the products obtained before testing them in an in vivo study.

## 2. Materials and Methods

### 2.1. Material

Brazil nuts (*Bertholletia excelsa* Bonpl.) were obtained from the Cooperativa Mista de Guariba (COMIGUA, Colniza, Mato Grosso, Brazil). Bocaiuva (*Acrocomia aculeata* (Jacq.) Lodd.) samples were obtained from rural producers (Poconé, Mato Grosso, Brazil) in 2016/2017 (SISGEN AE870E9). Both materials are coproducts of the food industry. Brazil nut coproducts came from separating whole Brazil nuts (material not selected for commercialization due to mechanical damage or a lack of homogeneity in size) [12]. Bocaiuva almond comes from fruit pulp extraction, whereby the pulp is extracted for food processing for consumption, in fresh form or as juices [15], and the almonds are always discarded. Brazil nut and bocaiuva almond were homogenized to have only one batch of each raw material. The calcium (Calcium Bisglycinate Chelate, code 03450) and iron (Ferrochel^®^ Ferrous Bisglycinate Chelate, code 03509) minerals were obtained in the form of chelating minerals (Albion^®^ Minerals, Clearfield, PA, USA).

All reagents used in this work have analytical standards. DPPH (2,2-diphenyl-1-picrylhydrazyl), Trolox (6-hydroxy-2,5,7,8-tetramethylchromane-2-carboxylic acid), ABTS (2,2-Azino-bis (3-ethylbenzothiazoline-6-sulfonic acid), Folin & Ciocalteu phenol reagent, and gallic acid were all purchased from Sigma Chemical Co. (St. Louis, MO, USA).

### 2.2. Preparation of Beverages

The Brazil nuts and bocaiuva almonds were sanitized (1:100, NaClO:H_2_O, *m*/*v*, for 15 min), and the edible part was manually separated. The beverages were prepared by grinding the nuts and almonds in distilled water at 50 °C/3 min, at the speed of 6000 *g*, using a RI 7625 blender (Phillips, Varginha, Brazil). The solid residue was separated using a cheesecloth (~100 mesh). All beverage formulations were prepared on the same day as the analysis and kept refrigerated until analysis (~7 °C).

Initially, we evaluated the changes in the beverages after heat treatment (HT) and compared them with beverages without HT (control, CT). Based on technological quality, HT was chosen and evaluated for the enrichment of the beverage with minerals in two concentrations (Figure S1). Finally, the effect of beverage administration on Wistar rats was evaluated. Five beverage formulations were developed as follows: (i) Brazil nut beverage (NB, nut:water = 1:10 m/v); (ii) bocaiuva almond beverage (AB, almond:water = 1:10 *m*/*v*); (iii) Brazil nut and bocaiuva almond beverage (NAB1, 1:0.1:10 = nut:almond:water; *m*/*m*/*v*); (iv) Brazil nut and bocaiuva almond beverage (NAB5, nut:almond:water = 1:0.5:10; *m*/*m*/*v*); and (v) Brazil nut and bocaiuva almond beverage (NAB10, nut: almond:water = 1:1:10; *m*/*m*/*v*).

In the case of heat treatment (HT), pasteurization was carried out by heating at 60 °C for 20 min, followed by cooling in a water bath at 6 °C for 15 min [16]. The beverages received added calcium and iron after HT at concentrations of 5% (25 mg of calcium and 0.35 mg of iron in 100 mL) and 50% (250 mg of calcium and 3.5 mg of iron for 100 mL) of the Brazilian Dietary Reference Intakes (DRIs) (1000 mg of calcium and 14 mg of iron) [17].

### 2.3. Characterization of Vegetable-Based Beverages

#### 2.3.1. Chemical Characterization

The composition of the beverages was estimated using established methods [18] as follows: moisture, lipid [19], protein (conversion factor of 5.46 to Brazil nut and 5.18 to bocaiuva almond, No. 991.20), ash, and carbohydrate (3,5-dinitrosalicylic acid method including the sample hydrolysis step with boiling 2N HCl, followed by cooling and neutralization with 2N NaOH) contents [20]. Calorific value was calculated using Atwater conversion factors (proteins × 4, lipids × 9, and carbohydrates × 4) [21]. Values of pH and soluble solids (SS) were determined using a digital pH meter and a refractometer, respectively. Titratable acidity (TA) was performed by titration with NaOH (3.9 g/L) and expressed as a percentage (acidity in molar percent solution, *v*/*m*).

The crude extract of beverages was prepared as previously described by Larrauri et al. [22] and was used in total phenolic content determined using the Folin–Ciocalteu method [23]. Gallic acid was used as the standard, and the results were expressed as mg gallic acid equivalent (GAE)/100 g of sample.

The crude extract was also used to determine the antioxidant capacity via DPPH [24] and ABTS [25] methods. The results from both assays were expressed as mM TEAC (Trolox equivalent antioxidant activity) per 100 g of sample.

#### 2.3.2. Technological Properties

Color analysis of the beverages was performed using a Color Quest II spectrophotometer (Hunter laboratory, Reston, VA, USA) with the CIE L*a*b* system with an observer angle 10° and illuminant D65. Chroma and hue values were calculated using the a* and b* parameters.

For sedimentation analysis, 2 g of sample (*Wo*) was placed in 2 mL microtubes and centrifuged at 1360× *g* for 20 min at 25 °C (SCILOGEX, D1008U, Rocky Hill, CT, USA). The supernatant was removed, and the final weight was determined (*Wf*). The sedimentation percentage (*w*/*w*) was calculated using Equation (1).

(1) $$Sedimentation=\frac{\left(Wf\right)}{Wo}\;\times \;100$$

Osmolality analysis was performed using the cryoscopic method of an electronic cryoscope (ITR, MK 540, Esteiro, Brazil) [26]. Freezing point conversion was obtained using the equation Δtc = Kc × m, where Δtc is the cryoscopic descent (the difference between the initial freezing temperature of the pure solvent and the initial freezing temperature of the solution), Kc is the water cryoscopic constant (1.86 °C/mol/kg), and m is the molal concentration of the solute (osmolality expressed in mOsm/kg solvent).

### 2.4. Microbiological Parameters

The count of coliforms at 35 °C and coliforms at 45 °C, as well as the presence of *Salmonella* sp. in 25 g, established for Brazilian legislation, were performed after 24 h of processing the plant extracts, following methodologies described by Compendium of Methods for the Microbiological Examination [27].

### 2.5. Biological Assay

Male Wistar rats weighing approximately 100 g (30 to 32 days old) were randomized into different groups. The rats were individually housed in metabolic cages in environmentally controlled rooms with 55% relative humidity, 22 ± 1 °C temperature, and 12 h of a light/dark cycle. They received water and an autoclaved pelleted commercial feed ad libitum. The pelleted commercial feed contained 23% protein and 4% lipids (information obtained from product packaging) (Labina^®^, Sao Paulo, Brazil). The Ethics Committee approved the experimental protocol of Animal Use at the Federal University of Mato Grosso (CEUA/UFMT no 23108.101756/2015-67).

The animals were divided into 5 experimental groups (n = 7 rats per group), as follows: control group (control) that received water; positive control group that received commercial soy beverage (soymilk containing 5.2 g of proteins, 3.1 g of lipids, and 4.8 g of carbohydrate per 200 mL); Brazil nut beverage group (NB); bocaiuva almond beverage group (AB); and Brazil nut and bocaiuva almond beverage group (1:1:10; nuts:almond:water; *m*/*m*/*v*). The beverages were standardized for protein content (1 g protein per 100 mL of beverage). Thus, the commercial soymilk was diluted 2.5× (4:6, extract:water, *v*/*v*), and the almond extract of bocaiuva was concentrated (3:10, fruit:water, *m*/*v*).

NB, AB, and NAB10 beverages were enriched with 25 mg of calcium and 0.35 mg of iron for 100 mL (5% DRIs of minerals). The beverages were administered via orogastric gavage to rats at a volume of 1 mL/100 g body weight (measured daily and considering the maximum gastric capacity of the animals) in a single dose (ranging from 1.0 to 3.0 mL approximately) every day for 28 days.

After the 28th day, the animals were sedated using CO_2_ inhalation at the end of the experimental period, decapitated, and exsanguinated. Tissues were removed, weighed, and stored at −80 °C to measure the lipid and glycogen content.

Blood samples were collected into tubes containing an anticoagulant (EDTA) to determine the glucose levels in plasma. Aspartate aminotransferase, alanine aminotransferase, and urea in plasma were determined using a colorimetric method using specific kits. The spectrophotometric method determined the concentration of triglycerides, total cholesterol, high-density lipoprotein (HDL), and low-density lipoprotein (LDL) cholesterol in serum.

Lipids from the liver and retroperitoneal adipose tissue were determined by gravimetric methods after chloroform–methanol (2:1) extraction according to the protocol followed by Folch et al. [28]. Hepatic glycogen levels were measured using the method used by Carroll et al. [29], measuring absorbance at 490 nm. Results were expressed in mg/g of tissue.

### 2.6. Statistical Analysis

The results of beverage characterization are expressed in terms of the mean ± standard deviation (SD), and the results of the biological assay are expressed in terms of the mean ± standard error (SE). Levene’s test for homogeneity was initially used to determine whether the data complied with the assumptions required for parametric analysis. The ANOVA test (one way) was used to measure the significance of the results in replicates, followed by Tukey’s post hoc test. The differences were considered significant at *p* < 0.05. The data were subjected to statistical analyses using GraphPad Prism 6 software (version 6.0, GraphPad Software Inc., San Diego, CA, USA).

## 3. Results and Discussion

### 3.1. Effect of Heat Treatment on the Characteristics of Vegetable-Based Beverages

**Figure 1:**
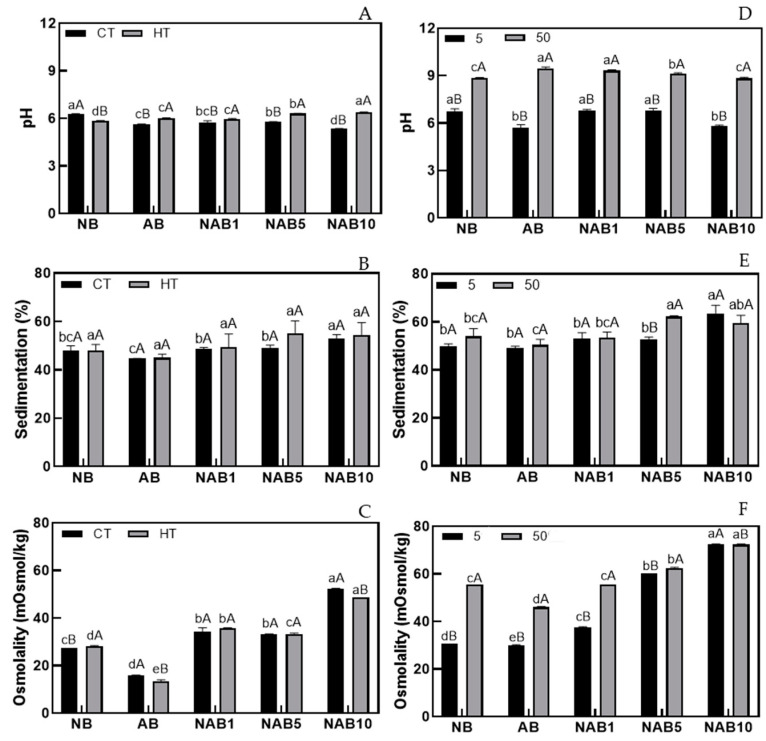
pH value, sedimentation (%), and osmolality (mOsm/kg) in beverages with Brazil nut and bocaiuva almond (CT) after heat treatment (HT) (**A**–**C**) and supplemented with 25 mg of calcium plus 0.35 mg of iron and 250 mg of calcium plus 3.5 mg of iron (5 and 50% DRIs of minerals, respectively) (**D**–**F**). Different lowercase letters indicate statistical differences between beverages with the same treatment (CT, HT, 5%, or 50%) according to Tukey’s test. Different capital letters indicate statistical significance between different treatments (CT and HT or 5% and 50%) in the same beverage (*p* < 0.05) according to Tukey’s test.

Figure 1 shows the impact of thermal treatment (HT) on pH (1A), sedimentation (1B), and osmolality (1C) of the beverages. The pH value ranged from 5.3 to 6.4 (Figure 1A). When HT was a step of beverage production, the pH value of all beverages increased, except for the NB treatment. On the other hand, the titratable acidity (ranging from 0.1 to 0.5%) showed no significant difference after HT treatment in any of the evaluated beverages (Table S1).

Although a low pH extends the shelf life of food products, and a pH lower than 4.5 is recommended to ensure microbiological safety [30], beverages with pHs < 5.5 are associated with demineralization of tooth enamel, and their consumption has been strongly discouraged, especially in children [31]. Furthermore, most commercial plant-based beverages have approximately the same pH, as reported by Frühauf et al. [32]. These authors evaluated commercial soy, cashew nut, oat, rice, and spelled grain beverages and found pHs of 5.82–7.42.

A lower percentage of sedimentation was found in AB treatment (CT and HT treatments) compared to other beverages evaluated, and adding bocaiuva almond to Brazil nut beverage increased the sedimentation. The results of soluble solids varied from 0.80 to 2.60 °Brix for AB and NAB10 treatments, respectively. Although the soluble solids demonstrated no significant difference for HT, these values corroborate the sedimentation values, indicating that greater sedimentation occurs when the beverages have higher soluble solids [32].

**Figure 2:**
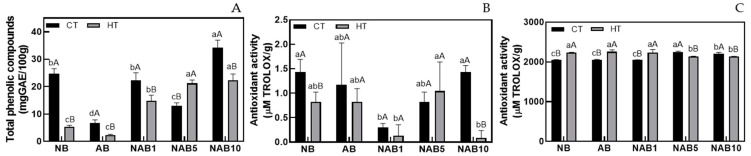
Total phenolic compounds (**A**) and antioxidant activity were measured using DPPH (**B**) and ABTS (**C**) methods in the bocaiuva almond and Brazil nut beverages. Different lowercase letters indicate statistical differences in similarly treated beverages (CT or HT), and different uppercase letters indicate statistical differences between CT and HT (*p* < 0.05) according to Tukey’s test.

Osmolality is used as a quality parameter for developing beverages for athletes. These beverages are classified as either hypotonic (osmolality < 270 mOsm/kg water), which promotes gastric emptying and water absorption from the proximal small intestine [33,34], or hypotonic (13 to 52 mOsmol/kg) (Figure 2C), such as the beverages (CT and HT). The osmolality of beverages was lower than that compared to fermented soymilk (170–500 mOsm/L) [35] and sports drinks (180–600 mOsm/L) [36].

Table S2 shows the color parameters of beverages. Luminosity (L*) parameters can indicate light (closer to 100) or dark (closer to 0) colors in the food, while the hue angle is defined using the angular scale of red (0°), yellow (90°), green (180°), and blue (270°). The saturation (Chroma) parameters and the intensity of the color define the colors as alive. The treatments showed higher luminosity with values between 50 and 65, with no significant difference between CT and HT, except for AB. This white behavior of substances has already been reported for other commercial plant-based beverages [32] and fermented plant-based beverages [15,35]. In contrast, beverages showed a green-yellowness (232–93°), with HT showing a significant decrease in the hue angle, compared to the CT beverages except for NAB1.

Figure 2 shows the total phenolic content (Figure 2A) and antioxidant activity measured by the DPPH (Figure 2B) and ABTS (Figure 2C) methods of beverages. The Brazil nut and bocaiuva almond showed 45.93 and 9.67 mg GAE/100 g of phenolic compounds. These values lower than 100 mg GAE/100 g classify Brazil nut and bocaiuva almond as having low TPC content according to the classification designated by Vasco et al. [37] and were close to other Cerrado fruits [38].

Not surprisingly, since the raw material was diluted in water, the beverages presented lower TPC than the raw material. However, phenolic compounds are more easily released from food matrices that are poor in dietary fiber, accessed during digestion, such as beverages, compared with the matrix and those linked to the dietary fiber, such as nuts and almonds [9].

TPC decreased when HT was used, except for NAB5. This demonstrates the thermosensitivity of the phenolic compounds present in the beverage, which has been reported in the literature [39].

The Brazil nut and bocaiuva almond showed 3.34 and 3.69 µM Trolox/g and 2097.68 and 2074.35 µM Trolox/g antioxidant activity using the DPPH and ABTS methods. Antioxidant activity from DPPH screening ranged from 0.1 to1.4 μM Trolox/g in all treatments evaluated (Figure 2B). There was a significant difference when heat treatment was used only in NB and NAB1, decreasing from 1.4 and 1.1 to 0.8 and 0.9 μM Trolox/g, respectively. In contrast, there was a significant difference in antioxidant activity for all beverages, as measured by the ABTS method, between the CT and HT beverages (Figure 2C).

The DPPH method measures the amount of DPPH scavenging by the antioxidant in polar organic media and may, therefore, be associated with the quantification of hydrophilic compounds. Meanwhile, the ABTS method measures the activity of hydrophilic and lipophilic compounds. Indirect methods used in the present work are considered practical and convenient and used for preliminary screening procedures [40,41].

Brazil nuts and bocaiuva almonds are rich in fatty acids [13,42] and can contribute to the antioxidant activity measured in the present study. The antioxidant activity significantly protects against oxidative stress even at low concentrations, in which free radicals are inactivated [43].

Microbiological evaluation of all developed beverages showed <3.0 and <10 CFU/mL for Coliform at 35 °C and 45 °C, respectively, and <10 CFU/mL for fungi and yeast, as well as the absence of *Salmonella* spp. per 25 g for all plant-based beverages. All microorganisms enumerated met the microbiological standards stipulated by Brazilian legislation. This indicates that all beverages are microbiologically safe.

Thermal treatment has long been used as a processing method to extend the shelf life of food products by eliminating or reducing spoilage and pathogenic microorganisms [8]. Thus, the present work performed HT in plant-based beverages to improve microbiological quality.

### 3.2. Effect of Mineral Supplementation on Characteristics of Beverages

Figure 1D–F shows the technological properties of plant-based beverages supplemented with minerals. There was a significant increase in pH from 4–5 to 9–10 in beverages with 25 mg of Ca plus 0.35 mg of Fe (5% DRIs of minerals) and 250 mg of Ca plus 3.5 mg of Fe (50% DRIs of minerals) treatments (Figure 1D), probably due to the neutralization of electric charges in protein molecules [8].

Surprisingly, adding minerals did not alter the sedimentation property of the developed beverages, except for NAB5 (Figure 1E). On the other hand, adding minerals increased the osmolality of the beverages. However, beverages can still be considered hypotonic (osmolality < 270 mOsm/kg).

The addition of minerals (regardless of concentration) decreased the luminosity parameter (except for NB and NAB1) and increased the hue angle (except for NAB5) (Supplementary Table S2). There was a reduction of approximately half the luminosity in the luminosity of the beverages and a change in tonality of the hue angle, which indicates red color (0° = red and 90° = yellow). Yellowness was observed for beverages with mineral supplementation for all samples (79° a 98°). The pigment saturation in the beverages was low (0.6 to 3.4) and therefore considered opaque.

Adding 25 mg of calcium plus 0.35 mg of iron (5% DRIs of minerals) demonstrates the potential of beverages, although this addition influences the technological properties (mainly pH and color). Mineral supplementation, particularly calcium and iron, is an interesting aspect of commercial beverages. Vitoria [44] evaluated 164 plant-based beverages containing calcium and/or vitamin D, and they found that more than half of the beverages had low calcium content.

### 3.3. Proximal Composition of Brazil Nut, Bocaiuva Almond, and Beverages

Table 1 shows the chemical composition of Brazil nut and bocaiuva almonds and the beverage developed in this study. The composition of the materials obtained in the present study was close to those reported in the literature for lipid (46–52 g/100 g) [14,45] and protein contents (17–24 g/100 g) [45] in bocaiuva almond, as well as higher lipid and protein contents (57–66 and 13 g/100 g, respectively) [43,46] in Brazil nut. The observed differences may be due to several biotic and abiotic factors that impact the chemical composition of raw materials.

Table 1 presents the chemical composition of the beverages’ submitted HT, supplemented with 25 mg of Ca plus 0.35 mg of Fe (5% DRIs of minerals). AB showed significantly higher moisture content, followed by NB and NAB1. Not surprisingly, NAB5 and NAB10 presented a lower moisture content, considering that these beverages have the highest amount of added bocaiuva almond. The formulations present no significant difference in ash content.

As occurred with Brazil nut and bocaiuva almonds, the lipid and protein contents of the beverages showed prominence over other constituents. NB presented a higher lipid content than other formulations developed. In addition, it was possible to notice that Brazil nuts contributed to reducing the lipid content of the beverages. The lipid content in beverages ranged from 5–17 g/100 g, which was higher than that reported for fermented soymilk (1.28–1.74 g/100 g) [35], as well as commercial rice, almond, oat, and coconut (0.9–2.0 g/100 mL) [44].

The protein content in AB was lower than that in other beverages. This value was close to what was reported for rice beverages [44], which are considered low-protein. It may cause Kwashiorkor, protein-energy malnutrition observed in infants on a rice-based vegan diet [7]. The combination of Brazil nut and bocaiuva almond can produce a high-protein beverage. It can circumvent this problem by increasing the protein value by approximately three-fold (1.20 g/100 g for NAB10).

Although AB had the lowest protein and carbohydrate contents among the beverages developed, this did not influence the reduction in these contents in NAB1, NAB5, and NAB10 since the developed formulations were prepared in proportion. The highest proportion corresponds to the greatest addition of solid material (Brazil nut + bocaiuva almond) in the beverages.

### 3.4. Effect of Beverages Administration on Wistar Rats

The present work evaluated if plant-based beverages administered to Wistar rats modified physical and biochemical parameters after 28 days. The control, soymilk, NB, AB, and NAB10 groups presented body weight averages of 183.3, 180.06, 175.04, 184.3, and 170.4 g, respectively, with no significant difference. This is an interesting result because biological assays usually show differences between negative control and treatment groups [47], demonstrating no difference even though the treatment groups ingested a higher protein content and other nutrients by administering the beverages.

**Figure 3:**
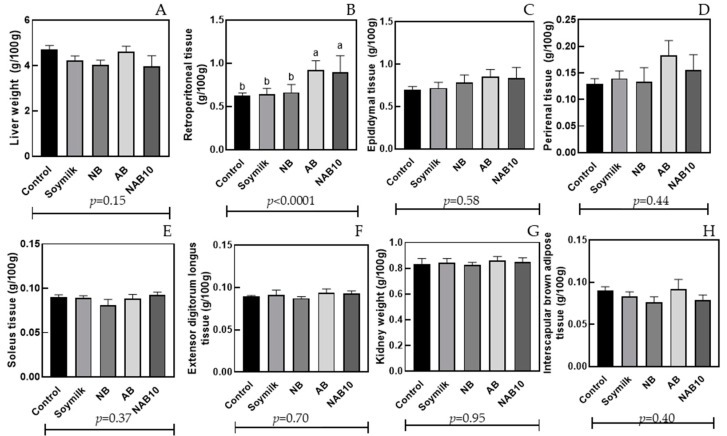
Liver weight (**A**), retroperitoneal tissue (**B**), epididymal tissue (**C**), perirenal tissue (**D**), soleus tissue (**E**), extensor digitorum longus tissue (**F**), kidney weight (**G**), and interscapular brown adipose (**H**) in Wistar rats after administering (28 days) beverages produced with Brazil nut and bocaiuva almond and supplemented with 25 mg of calcium plus 0.35 mg of iron (5% DRIs of minerals). Different letters indicate statistical differences according to one-way ANOVA between the groups evaluated (*p* < 0.05).

Figure 3 shows the weight gain in the animal organs. The liver weight ranged from 4.02 g/100 g for NB to 4.71 g/100 g for the control group, with no significant difference (Figure 3A). All beverages evaluated showed a lower liver weight than the control group, emphasizing NB and NAB10 beverages (*p* = 0.26). In contrast, Jaekel and Rodrigues [48] showed a higher liver weight/body weight in the liver of hamsters fed soymilk (4.97 g/100 g) than in the control treatment. The high weight of the liver indicates an accumulation of lipid contents, which may indicate Nonalcoholic Fatty Liver Disease or Nonalcoholic Steatohepatitis (NAFLD or NASH, respectively). Dietary components play an important role in the development and progression of NAFLD/NASH. Further evidence now suggests that the diet’s composition, particularly lipid and carbohydrate contents, also plays an essential role in disease progression to NASH and fibrosis [49].

Epididymal tissue, perirenal tissue, soleus tissue, extensor digitorum longus tissue, kidney weight, and interscapular brown adipose (Figure 3C–H) demonstrated no significant difference between groups. Only the retroperitoneal adipose tissue among the evaluated organs and tissues showed significant differences between the treatments. Control, soymilk, and NB (0.62, 0.64, and 0.66 g/100 g, respectively) showed no significant difference. In comparison, AB and NAB10 (0.92 and 0.89 g/100 g, respectively) showed higher values with significant differences compared to the other treatments (*p* < 0.0001) (Figure 3B). A high retroperitoneal adipose tissue weight was found in groups of Wistar rats where bocaiuva almond-containing beverages were administered. This behavior may be due to this almond’s high saturated fatty acid content (33 g/100 g), resulting in a ratio of ~1 compared to unsaturated fatty acid content [42]. A high ratio of unsaturated fatty acids to saturated fatty acid content is associated with improved human health [50].

**Figure 4:**
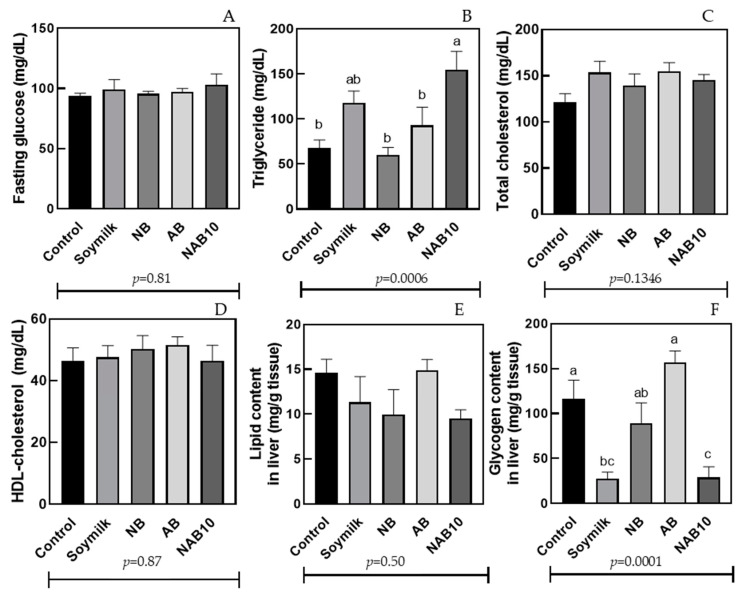
Fasting glucose (**A**), triglyceride (**B**), total cholesterol (**C**), HDL cholesterol (**D**), lipid content in liver (**E**), and glycogen content in liver (**F**) of Wistar rats after administering (28 days) beverages produced with Brazil nut and bocaiuva almond and supplemented with 25 mg of calcium plus 0.35 mg of iron (5% DRIs of minerals). Different letters indicate statistical differences according to one-way ANOVA between the groups evaluated (*p* < 0.05).

Figure 4 shows the biochemical parameters in the blood and liver of animals ingesting beverages. The fasting glucose ranged from 93 to 103 mg/dL and showed no significant difference between the animals receiving the beverages. The total cholesterol (97–170 mg/mL) (Figure 4C) and HDL cholesterol (46–52 mg/mL) (Figure 4D) showed no significant differences between the animals receiving the beverages. The total cholesterol showed a slight increase in soymilk (153.66 mg/dL) and AB (154.82 mg/dL) groups compared with the control group (121.22 mg/dL) (*p* = 0.16 and *p* = 0.14, respectively). In contrast, higher triglyceride contents were found in NAB10-fed mice (154.8 mg/dL) with no significant difference with soymilk (117.8 mg/dL). The NB- (59.9 mg/dL) and AB (92.8 mg/dL)-fed mice showed no difference from the control group (68 mg/dL) (Figure 4B) for triglyceride values.

A different result was presented by Ajiboye et al. [51], who administered fermented sorghum/millet-based beverages to rats for 28 days and showed that there was no significant difference in the lipid profile (total cholesterol, total triglycerides, and HDL-c) of the control and beverage groups.

Bocaiuva almond has a higher fatty acid content, containing oleic acid (37 g/100 g), followed by lauric acid (33 g/100 g) [13]. On the other hand, Brazil nuts have the highest amount of linoleic acid (38 g/100 g), followed by oleic acid (32 g/100 g) [52]. Oleic acid is the most potent fatty acid associated with increased Apo B secretion, followed by linoleic acid [53]. Consequently, Apo B inhibits lipase activity, which is responsible for the transport of triglycerides into the bloodstream [54]. The lipase hydrolyzes dietary triglyceride molecules to glycerol and fatty acids, which then bind to albumin for transportation into the bloodstream as an energy source. In contrast, the blood releases and carries glycerol freely to the liver [55]. The effect of lipid quality was only observed in the NB group, where its high lipid content (Table 1) and lipid quality [11] resulted in lower total triglycerides and retroperitoneal tissue weight.

The liver lipid content showed no statistical difference between the treatments administered to Wistar rats (Figure 4E). All fed groups, except for AB, had a lower lipid content than the control group. This result corroborates the slightly lower liver weight values (Figure 4). The effect of lipid quality was only observed in the NB group, where its high lipid content (Table 1) and lipid quality [11] resulted in lower total triglycerides and retroperitoneal adipose tissue weight.

The glycogen content in the liver was higher in group AB- and NB-fed mice (Figure 4F). Liver glycogen accumulation is important as it is the source of new energy for the body between meals. In addition, studies suggest that stellate liver cells control blood cholesterol levels by transporting excess fat to the liver [56]. This behavior seems to have occurred in the NAB10 group, which demonstrated low liver glycogen and higher total triglycerides than the AB group.

The aspartate aminotransferase, alanine aminotransferase, total protein, and creatinine contents showed no significant difference between the groups (Table 2).

The soymilk group showed the highest urea content compared to the other treatments. In this study, the protein in the diet was controlled by the administration of the beverage, but it is known that soymilk contains proteins with high bioavailability. These nitrogenous compounds obtained from proteins are metabolized to form ammonia. Ammonia metabolites are directed to the liver to synthesize urea and nitrogenous waste, which the kidneys eliminate [56]. The measurement of urea is used in early examinations of renal insufficiency because it demonstrates changes before the creatinine measurement (as occurred in the present study).

Hu et al. [57] administered six Brazil nuts to human volunteers (>50 years old) and reported a significant increase in urea levels in subjects consuming Brazil nuts. In the present work, we found a decrease in urea levels for the beverage produced with Brazil nut compared to control-fed (without significance) and soymilk-fed groups (with significance).

To our knowledge, this is the first study that evaluated the beverage intake of Brazil nut and bocaiuva almond enriched with 25 mg of Ca plus 0.35 mg of Fe (5% DRIs of minerals). The beverages evaluated in this study demonstrate a protective effect against risk factors for metabolic syndrome, especially the fat accumulation in the liver (hepatic steatosis), retroperitoneal adipose tissue accumulation, and the hypercholesterolemic effect. The Brazil nut beverage decreased retroperitoneal adipose tissue, total cholesterol, and triglycerides. The bocaiuva almond beverage decreased triglyceride contents. Meanwhile, the combination of Brazil nut and bocaiuva almond decreased liver weight.

## 4. Conclusions

Considering that microbiological quality must be guaranteed, plant-based beverages obtained from Brazil nut and bocaiuva almond via heat treatment at 60 °C for 20 min demonstrated the best stability.

Adding 5% DRI minerals (25 mg of calcium and 0.35 mg of iron) improves the nutritional value of beverages with Brazil nut and bocaiuva almond. However, this mineral addition influences beverages’ technological properties (mainly the pH and color). Furthermore, the combination of Brazil nut and bocaiuva almond can produce a higher-protein beverage.

Wistar rats demonstrated physical (increased retroperitoneal adipose tissue weight) and biochemical (total triglyceride and hepatic glycogen content) changes when administered a daily portion of the beverage for 28 days. Other animal models must be tested to validate these results, including female animals and obesogenic models. Furthermore, like any animal study, this study has the limitation that its conclusions only suggest a trend, and its extrapolation to humans must include randomized controlled trials.

This study demonstrated that using bocaiuva almond is viable in developing new products and contributing to nutritional and biochemical aspects, along with Brazil nuts. Further studies are still needed to conclude that these beneficial effects on human health can be associated with consuming the developed beverage. Still, to our knowledge, this manuscript reports for the first time the use of these two oilseeds in developing food products with potential beneficial effects on health.

## Figures and Tables

**Table 1:** Proximal composition (g/100 g) of Brazil nut, bocaiuva almond, and beverages produced with Brazil nut and bocaiuva almond and supplemented with 25 mg of calcium plus 0.35 mg of iron (5% DRIs of minerals). Different letters in the same column indicate statistical significance (*p* <0.05) according to Tukey’s test. Brazil nut × bocaiuva almond and beverages × beverages.

	Moisture	Ash	Lipids	Protein	Carbohydrate
Brazil nut	3.00 ± 0.20 ^a^	2.90 ± 0.20 ^a^	74.00 ± 5.00 ^a^	16.70 ± 0.30 ^a^	6.00 ± 2.00 ^a^
Bocaiuva almond	15.00 ± 0.10 ^b^	1.50 ± 0.20 ^b^	42.50 ± 0.50 ^b^	16.20 ± 0.20 ^b^	24.70 ± 0.30 ^b^
NB	93.20 ± 0.60 ^b^	0.12 ± 0.08 ^a^	17.00 ± 10.00 ^a^	1.05 ± 0.02 ^a^	0.41 ± 0.03 ^a^
AB	95.40 ± 0.60 ^a^	0.12 ± 0.03 ^a^	5.00 ± 4.00 ^b^	0.44 ± 0.01 ^c^	0.26 ±0.02 ^b^
NAB1	92.70 ± 0.30 ^b^	0.07 ± 0.05 ^a^	9.00 ± 1.00 ^b^	1.05 ± 0.07 ^a^	0.50 ± 0.01 ^a^
NAB5	89.50 ± 0.40 ^c^	0.20 ± 0.10 ^a^	7.00 ± 1.00 ^b^	0.82 ± 0.08 ^b^	0.40 ± 0.02 ^a^
NAB10	89.80 ± 0.20 ^c^	0.30 ± 0.20 ^a^	6.80 ± 0.50 ^b^	1.20 ± 0.10 ^a^	0.65 ± 0.04 ^c^

**Table 2:** Biochemical parameters of Wistar rats after administering (28 days) beverages produced with Brazil nut and bocaiuva almond and supplemented with 25 mg of calcium plus 0.35 mg of iron (5% DRIs of minerals). Different letters on the same line indicate statistical differences according to one-way ANOVA between the groups evaluated (*p* < 0.05).

Parameters (mg/dL)	Control	Soymilk	NB	AB	NAB10	*p*-Value
Aspartate aminotransferase	15.40 ± 5.20 ^a^	15.50 ± 2.10 ^a^	14.40 ± 4.00 ^a^	18.40 ± 3.80 ^a^	15.20 ± 8.50 ^a^	0.66
Alanine Aminotransferase	80.81 ± 9.60 ^a^	82.80 ± 13.00 ^a^	75.52 ± 18.80 ^a^	88.40 ± 15.40 ^a^	79.30 ± 11. 20 ^a^	0.53
Urea	68.00 ± 11.30 ^a^	113.10 ± 35.70 ^b^	64.90 ± 12.20 ^a^	65.80 ± 17.70 ^a^	72.90 ± 13.50 ^a^	0.00
Total protein	5.70 ± 0.3 ^a^	5.80 ± 0.50 ^a^	6.00 ± 0.50 ^a^	5.80 ± 0.60 ^a^	5.46 ± 0.50 ^a^	0.39
Creatinine	0.21 ± 0.00 ^a^	0.28 ± 0.00 ^a^	0.28 ± 0.00 ^a^	0.25 ± 0.00 ^a^	0.27 ± 0.00 ^a^	0.28

## Data Availability

The original contributions presented in the study are included in the article/Supplementary Material, further inquiries can be directed to the corresponding authors.

## Supplementary Materials

The following supporting information can be downloaded at: https://www.mdpi.com/article/10.3390/foods13162533/s1, Figure S1: Flowchart of development of Brazil nut beverage supplemented with bocaiuva almond and minerals; Table S1: Titratable acidity (%, *v*/*m*) parameters of beverages; Table S2: Color parameters of plant-based beverages.
