# Supplementary material for: Beverage of Brazil Nut and Bocaiuva Almond Enriched with Minerals: Technological Quality and Nutritional Effect in Male Wistar Rats
Source: Foods. 2024 Aug 14;13(16):2533. doi: 10.3390/foods13162533 (PMC11353289; doi:10.3390/foods13162533)
Supplement: Supplementary file 1 [file foods-13-02533-s001.zip › foods-3049041-supplementary.pdf]

Supplementary Figure S1. Flowchart of development of Brazil nut beverage supplemented with bocaiuva almond and minerals

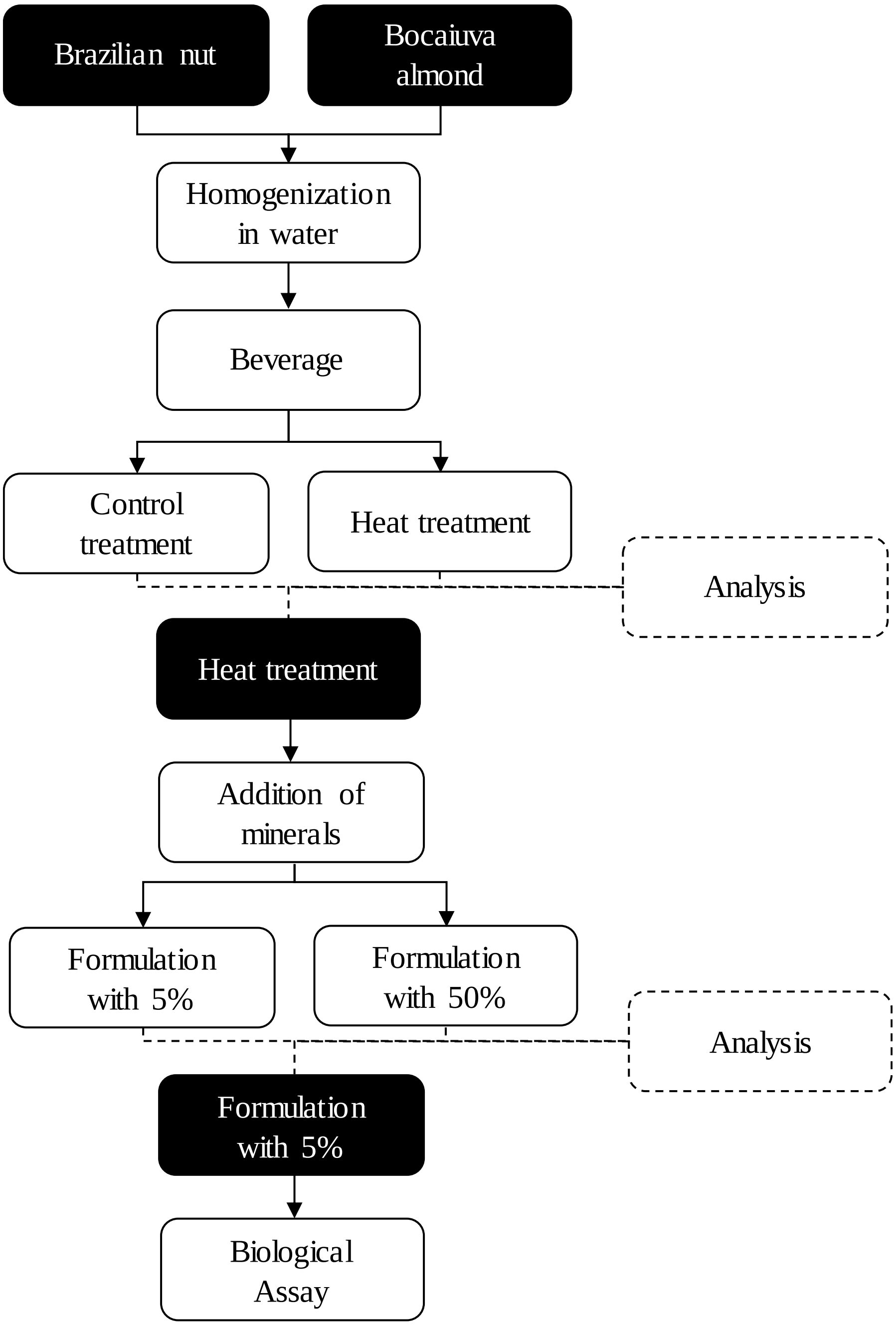

Supplementary Table S1. Titratable acidity (%, v/m) parameters of beverages. Different lower-case letters indicate statistical differences between beverages undergoing the same treatment (CT or HT). Different capital letters indicate statistical differences between different treatments (CT or HT) in the same beverages (*p*<0.05).

		Titratable acidity (%)
NB	CT	0.17 ± 0.07^{aA}
	HT	0.30 ± 0.13^{abA}
AB	CT	0.22 ± 0.07^{aA}
	HT	0.19 ± 0.07^{aA}
NAB1	CT	0.30 ± 0.07^{aA}
	HT	0.19 ± 0.07^{aA}
NAB5	CT	0.22 ± 0.07^{aA}
	HT	0.26 ± 0.07^{abA}
NAB10	CT	0.35 ± 0.07^{aA}
	HT	$0.46 \pm 0{,}07^{bA}$

Supplementary Table S2. Color parameters of plant-based beverages. Different lower-case letters indicate statistical differences between extracts undergoing the same treatment: CT, HT, 5%, or 50%. Different capital letters indicate statistical differences between different treatments (CT and HT or 5% and 50%) in the same extract (p<0.05).

		L*	°h	Chroma
		Heat treatment		
NB	CT	64.08±6.55^{bA}	154.04±5.63^{cA}	1.44±0.07^{aA}
	HT	62.44±6.00^{aA}	118.22±4.51^{bB}	3.03±0.03^{cB}
AB	CT	38.96±2.08^{aA}	232.45±4.74^{dA}	1.39±0.47^{aA}
	HT	56.59±5.37^{aB}	142.31±1.89^{cB}	0.66±0.05^{aB}
NAB1	CT	53.50±2.31^{bA}	137.85±4.83^{bA}	1.55±0.03^{aA}
	HT	55.94±6.07^{aA}	126.36±7.80^{bA}	1.78±0.05^{bA}
NAB5	CT	60.25±6.70^{bA}	121.41±4.51^{aA}	1.90±0.05^{aA}
	HT	64.92±6.61^{aA}	104.24±2.36^{aB}	2.73±0.05^{dB}
NAB10	CT	64.82±6.98^{bA}	114.89±3.93^{aA}	3.36±0.14^{bA}
	HT	62.74±5.95^{aA}	93.51±1.91^{aB}	3.00±0.03^{eA}
		Mineral Supplementation		
NB	5%	27.34±1.16^{aA}	98.53±0.82^{aA}	2.96±0.25^{aA}
	50%	27.09±0.72^{aA}	88.77±0.34^{aB}	4.77±0.64^{aB}
AB	5%	24.58±0.13^{bA}	92.61±0.37^{bA}	3.34±0.01^{abA}
	50%	19.37±0.18^{bB}	79.73±0.39^{bdB}	4.16±0.05^{aB}
NAB1	5%	28.55±0.28^{aA}	91.85±0.07^{bA}	3.68±0.05^{bcA}
	50%	23.62±0.11^{cB}	89.50±0.23^{aA}	4.72±0.03^{aB}
NAB5	5%	26.12±0.31^{aA}	90.48±0.45^{bA}	3.77±0.03^{bcA}
	50%	23.70±0.16^{cB}	86.85±0.33^{cB}	4.29±0.04^{aA}
NAB10	5%	24.65±0.05^{bA}	91.15±0.68^{bA}	3.99±0.02^{cA}
	50%	23.91±0.08^{cA}	79.83±0.05^{dB}	2.46±0.04^{bB}
